# Discriminatory Practices in the German Mental Healthcare System: An Intersectional Grounded Theory Study

**DOI:** 10.1177/10497323251325793

**Published:** 2025-05-15

**Authors:** Neneh Rosalía Quadflieg, Patiani Batchati, Alva Träbert, Eike Leidgens, Georg Juckel, Jakov Gather, Amma Yeboah, Mirjam Faissner

**Affiliations:** 1 Department of Psychiatry, Psychotherapy and Preventive Medicine, LWL University Hospital, 9142 Ruhr University Bochum, Bochum, Germany; 2 Rassismuskritische Psychotherapie e.V., Berlin, Germany; 3 German Association of Psychosocial Centers for Refugees and Victims of Torture, Berlin, Germany; 4 Rosa Strippe e.V., Bochum, Germany; 5 Medical Care Service for Refugees Bochum, Bochum, Germany; 6 Institute for Medical Ethics and History of Medicine, 9142 Ruhr University Bochum, Bochum, Germany; 7 Private Practice for Psychiatry and Psychotherapy, and Psychodynamic Supervision, Cologne, Germany; 8 Institute of the History of Medicine and Ethics in Medicine, 14903 Charité - Universitätsmedizin Berlin, Berlin, Germany

**Keywords:** racism, intersectionality, LGBTQIA, trans* health, healthcare access, minority groups, structural discrimination, psychiatry

## Abstract

Discriminatory practices within mental healthcare are a major barrier to the equitable provision of care to *all* mental healthcare service users. Understanding mechanisms of discrimination is a prerequisite for developing suitable measures to address them. Intersectionality, a framework rooted in Black feminism, has proven to be a powerful tool for understanding the specific forms and experiences of discrimination within interconnected systems of oppression. This study is the first to use an intersectional lens to examine discriminatory practices within the German mental healthcare system from the perspectives of service users, providers, and psychosocial counselors. In collaboration with local organizations in Bochum, Germany, we conducted 17 semi-structured interviews. The data were analyzed according to constructed grounded theory methodology. Our results indicate that discriminatory practices undermine access to and quality of healthcare delivery for marginalized mental healthcare service users. On an interpersonal level, these practices included stereotyping, devaluation, Othering, invalidation, silencing, withholding information, and treatment refusals. On an organizational level, care was undermined by a lack of interpretation services, discriminatory admission practices and documentation procedures, a lack of competencies among mental healthcare providers, as well as suitable treatment options and environments for marginalized service users. Service users described various strategies to navigate mental healthcare, including confrontation and selective narration. Mental healthcare providers showed various reactions toward discriminatory practices, ranging from defensiveness to acknowledgment. We discuss the results in their interrelationship with institutional Whiteness, cis-heteronormativity, and mental illness.

## Background

Health is a core element of human well-being and the prerequisite for individuals to develop and exercise their capacities (Powers & Faden, 2019). It is, therefore, a matter of social justice that healthcare services are delivered free from discrimination. Discrimination is defined as a person’s differential treatment based on their social group membership, which structures interactions across various social contexts, for example, racialization, gender, sexual orientation, or ability (Lippert-Rasmussen, 2018). Regarding mental healthcare, research indicates that discrimination of marginalized^1^ service users occurs on multiple levels, including the interpersonal (e.g., bias, prejudice, and stereotyping in provider–service user interactions), institutional (e.g., standard operating procedures and representation of marginalized groups in research), and structural levels (disadvantages in the interaction between different systems, e.g., housing, healthcare, and education) (Schouler-Ocak et al., 2021). Discriminatory practices within healthcare are associated with a reduced quality of care, may lead to a loss of trust in the healthcare system, and more frequent crisis-related hospital admissions (DeZIM, 2023; Elias & Paradies, 2021; Hamed et al., 2022; Henderson et al., 2015). While critical research on racism is growing internationally, the German Psychiatric Association stresses the need for more research on discriminatory practices in German mental healthcare (DGPPN, 2020).

### Intersectionality in Mental Healthcare Research

Intersectionality has emerged as a crucial paradigm for understanding discriminatory practices. It is rooted in a long tradition of Black^2^ feminist thought (Hill Collins, 2000) and activism (Combahee River Collective, 2018) which goes back to slavery and prison abolition activists, like Sojourner Truth (2019). The term “intersectionality,” introduced by legal theorist Kimberlé Crenshaw, illustrates the way in which focusing on a single form of discrimination, such as racism, prevents a more complete understanding of the experiences of those who face multiple types of discrimination, such as heterosexism, racism, and classism (Crenshaw, 1989). It offers a lens to understand specific experiences of discrimination in relationship to structural conditions of social injustice, and can serve as an analytical and methodological tool within research projects (Hill Collins, 2015). The concept has been applied to various research fields, including epidemiological health research (Bauer, 2014) and psychology (Cole, 2009) in both quantitative (Else-Quest & Hyde, 2016) and qualitative research designs (Abrams et al., 2020). Some scholars have criticized the broad use of intersectionality in research if it does not live up to its theoretical commitments, for example, by failing to center Black women and queer perspectives, thus undermining its political potential (Bilge, 2013; Rice et al., 2019).^3^

### State of Research

Intersectionality has been applied to epidemiological research on mental health, highlighting the importance of intersectional approaches in analyzing mental health disparities (Fagrell Trygg et al., 2019; Patil et al., 2018). In terms of qualitative research, according to a recent systematic review, mental healthcare service users assumed factors related to their intersectional social identity, for example, as a Black trans person, to be highly relevant to their mental health, therapy experience, and access to services (Hempeler et al., 2024). Therefore, they considered their mental healthcare providers’ sensitivity to their social identity as crucial for therapy success. The review identified various mechanisms of discrimination on an interpersonal (e.g., invalidations, insults, treatment refusals, and silencing) and institutional level (e.g., lack of competencies regarding structural discrimination, admission procedures, and documentation systems) that reduce the quality of care for marginalized users.

Research on mental healthcare that takes an intersectional approach has been conducted primarily in the United States and Canada, leaving Germany with a gap in both quantitative and qualitative intersectional mental healthcare research. Regarding healthcare for the lesbian, gay, bisexual, trans, queer/questioning, inter, and asexual (LGBTQIA+) community, discriminatory practices in healthcare find mention, yet intersectional experiences are not given due attention (Kahl et al., 2022; Nieder et al., 2020). The German discourse on structural racism has long focused on mental healthcare for refugees and migrants through the lens of intercultural psychiatry (Kluge et al., 2020; Yeboah, 2017), with a few exceptions (e.g., Kilomba, 2010). In this context, the publication of the Afrozensus, a community-based participatory study, represents a significant contribution to the field of study, seeking to address the research gap on anti-Black racism in Germany (Aikins et al., 2021). Additionally, a new interdisciplinary research field on structural racism in Germany is emerging, supported by funding for a national survey on discrimination and racism in Germany (NaDiRa) including healthcare settings (DeZIM, 2023).

In order to address the dearth of research, this study aims to explore discriminatory practices in mental healthcare settings in Germany. We use an intersectional lens on discrimination, paying particular attention to the intersections of racism, cis-heteronormativity, and mental illness discrimination.^4^ Our research centers on the following questions: (1) Which discriminatory practices do service users, psychosocial counselors, and mental healthcare providers describe in mental healthcare? (2) How do service users and mental healthcare providers reportedly respond to such discriminatory practices, and which strategies do they describe to navigate or work in mental healthcare? (3) How do participants experience the impact of discriminatory practices on the quality of treatment and the mental health of those affected?

## Methods

### Study Design

We conducted a qualitative interview study as a collaboration between the Department of Psychiatry, Psychotherapy and Preventive Medicine at Ruhr University Bochum; the Institute for Medical Ethics and History of Medicine at Ruhr University Bochum; *Rosa Strippe e.V*., a psychosocial counseling center for LGBTQIA+ people; and *Medizinische Flüchtlingshilfe Bochum e.V*. (*MFH*), a psychosocial center for trauma therapy serving survivors of torture and war based in Bochum, Germany. As participatory research approaches are recommended for mental health research with marginalized groups (Stacciarini et al., 2011), we combined a collaborative study team with methods of participatory data analysis. A study team was formed for study coordination, consisting of three academic researchers (NRQ, PB, and MF) and two coresearchers with expertise in anti-discrimination and psychosocial counseling (EL and AT). All questions regarding the study design, sampling, and data analysis were jointly decided based on consensus in regular *jour fixe* meetings held first on a biweekly then monthly basis. Intersectionality informed and guided all stages of research (Abrams et al., 2020).

### Participants Sampling

Inclusion criteria for all participants were full legal age and decision-making capacity regarding research participation. We included (1) psychiatric service users with a self-reported mental health diagnosis and reported experiences of discrimination within mental healthcare, (2) mental healthcare providers, and (3) psychosocial counselors with expertise in counseling for marginalized service users. We used a theoretical sampling method to achieve diversity for the intersections of mental health diagnoses, treatment experiences, gender, racialization, sexual orientation, ability, and socioeconomic status, as well as professions.

### Recruitment

Psychosocial counselors were contacted via email sent to different local psychosocial community centers. Service users and mental healthcare providers were recruited through the Department of Psychiatry, Psychotherapy and Preventive Medicine, *Rosa Strippe*, and *MFH* via leaflets in German, English, French, and Turkish, outlining the study, the research team, the funding body, and information on the expense allowance. An invitation to participate in the study was circulated via email among the psychiatry network of the Regional Association of Westphalia Lippe (*PsychiatrieVerbund des Landschaftsverbands Westfalen-Lippe*), a large mental healthcare provider. Interested people contacted the last author via telephone or email. Every participant received written and verbal information about the study and provided their written informed consent to participate in the study. The participants were informed that they could receive mental healthcare support if they experienced distress during or after the interview. The study was approved by the Research Ethics Committee of the Medical Faculty of the Ruhr University Bochum (registration number 22-7504).

### Participant Characteristics

We included three psychosocial counselors, seven mental healthcare providers, and seven service users in the study. Table 1 displays information on the sociodemographic characteristics of the study participants. Among mental healthcare providers, four doctors (three psychiatric residents, one psychiatric consultant), one psychological therapist, and two psychiatric nurses, with a mean professional experience of 24 years (SD = 15), participated. The psychiatric diagnoses of the service users represented a wide range of F-diagnoses according to the ICD-10, with depressive disorder being the most common, followed by anxiety disorders. The time of initial diagnosis varied between 2015 and 2022, the number of in-patient psychiatric treatments ranging between one and four, and four participants had experiences with outpatient psychiatric treatment and/or psychotherapy.

**Table 1.** Sociodemographic Characteristics of the Study Participants: Mental Healthcare Service Users (*n* = 7), Mental Healthcare Providers (*n* = 7), and Psychosocial Counselors (*n* = 3).

Variable (Self-Description)	*N*_service user_	*N*_providers_	*N*_counselor_	*N*_total_	%
Age (M in years, SD in brackets)	30 (7)	47 (13)	46 (15)	41 (10)	
Gender identity					
Woman	1	3		4	24
Man	3	4	2	9	53
Trans woman	1			1	6
Trans*	3			3	18
Nonbinary	2		1	3	18
Rejection of gender categories			1	1	6
Racialization					
Person of color	1	2		3	18
Arab	2			2	12
Black		1		1	6
Latinx		1		1	6
*White*	4	5	3	12	71
Russian-German		1		1	6
Disability					
No	3	6	3	12	71
Yes	4			4	24
Perceptible to others					
Imperceptible to others	4			4	100
No information/would not like to comment		1		1	6
Sexual orientation					
Homosexual	1	2		3	18
Bisexual		1		1	6
Asexual	1			1	6
Pansexual	1	1		2	12
Queer	4			4	24
Heterosexual		3		3	18
Androphilic	1			1	6
No information/would not like to comment	1		3	4	24

*Note.* The sum of the percentages does not add up to 100% due to multiple answers.

### Data Collection

We conducted 17 semi-structured interviews between May 2022 and June 2023. Participants completed a sociodemographic survey on their intersectional social position and mental health status. The interviews were held in the localities of the *Rosa Strippe*, the *MFH*, the Department of Psychiatry, Psychotherapy and Preventive Medicine, or online via Zoom, based on participant ability and preference. The interviews were conducted by tandems of two researchers each, with the last author being present for all interviews. One interview was conducted in Arabic with a professional interpreter. The mean duration of the interviews was 59 minutes (SD = 14 minutes).

We first conducted expert interviews with psychosocial counselors to gather information about discriminatory practices within mental healthcare. Information from these interviews informed the revision of the interview guides for service users and mental healthcare providers. We employed problem-centered interviews (Witzel, 2000), asking about both positive and negative mental healthcare experiences, discrimination within mental healthcare, and their strategies to address discrimination. The interview guides contained prompts, open questions, and suggestions for concretizing and evaluating questions (see Online Appendix 1). The guides were handled freely and adapted to the conversations. Service users received a financial compensation of €25 for the interview itself and €15 for a member checking conversation. All interviews were audio-recorded, transcribed verbatim, and pseudonymized by an external transcription service. Selected quotes were translated from German to English by the last author and proofread by a bilingual speaker (AT).

### Data Analysis

We analyzed data in a constructed grounded theory approach according to Charmaz (2006) using MAXQDA 2022 Standard (VERBI Software GmbH, Berlin, Germany). Constructed grounded theory is congruent with intersectional theory in building on feminist epistemology that stresses the social situatedness of knowledge, requiring the joint investigation of social experiences by differently positioned individuals (Kassam et al., 2020). Additionally, in accordance with intersectionality, constructed grounded theory pays particular attention to the influence of power relations on social experiences and their reconstruction. After the initial interviews, NRQ, PB, and MF started the open coding process, inductively coding the transcripts using a sentence-by-sentence technique to analyze, break down, and categorize interview sections. Codes were compared, correlated, and classified within the different interviews. In the following axial coding, NRQ, PB, and MF developed categories as key focal points during an iterative coding process across the interviews, comparing the inductive categories to theoretical background assumptions. The researchers focused on interviews and categories in the axial coding that they had not initially coded, to account for intersubjective traceability. Six participatory data analysis sessions were organized with the study team and participants in which the axial categories were discussed, paying particular attention to the influence of interrelated systems of oppression. Additionally, service users and mental healthcare providers were invited to participate in member checking conversations, in which they were presented with the main categories emerging from their interview and invited to give feedback. Three out of seven service users and five out of seven mental healthcare providers participated in the member checking. NRQ, PB, and MF theorized relationships between categories, combining selective coding with considerations around intersectionality, and reassessed the research questions in the iterative process. Together with AY, the study team developed a conceptual model of discriminatory practices within mental healthcare, informed by counseling experiences with marginalized service users and using intersectionality to account for the complexity of social experiences in mental healthcare. The model was discussed with all authors to incorporate interprofessional perspectives from clinical practice, research, and anti-discrimination services.

### Study Team Characteristics and Researcher Reflexivity

The study team included the principal investigator (researcher in medical ethics), one M.A. student of psychology, one medical resident, and two counselors from the collaborating local community centers. The principal investigator (MF) has performed and supervised various qualitative research projects (Faissner et al. 2023, 2024; Hempeler et al., 2024), and all researchers involved in the data analysis received qualitative methods training by the Methods Centre of Ruhr University Bochum. In order to recognize power relations within the study team and toward participants, the study team jointly reflected on their social positions. The study team included one person identified as Black, one as a person of color (POC) and Latinx, and three as *white*. Several members identified as queer and some had experiences with mental healthcare services or psychotherapy. The composition of the study team and the collaboration with anti-discrimination experts facilitated mutual learning within the study team. Additionally, study participants reported that they felt more comfortable sharing painful experiences and were more confident that they would be met with understanding because marginalized social identities were represented among the interviewers. The study was supervised by a Black, cis-female psychiatric psychotherapist and specialist, and two *white* cis-male psychiatrists, allowing the study team to reflect their biases during data analysis, especially in terms of institutional Whiteness, as well as their attitude toward psychiatry as an institution.

## Results

**Figure 1.**
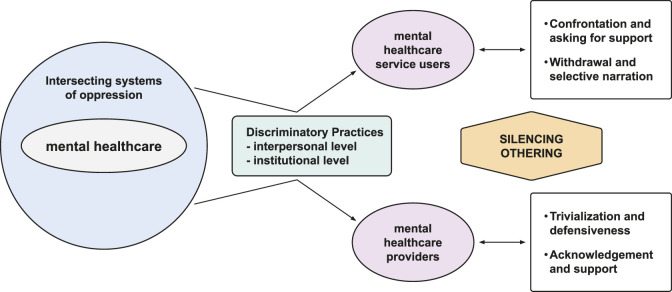
Relationship between themes and subthemes.

Three main themes and 15 subthemes were identified (see Table 2). Figure 1 illustrates the relationship between the themes. The first and last authors translated selected verbatim quotes from the interviews from German into English and inserted them in italics. Definitions of the subthemes and further exemplary quotes can be consulted in the Online Appendix 2. Salient social identities of the interviewees are indicated in brackets.

**Table 2.** Main Themes and Subthemes.

Main Themes	Subthemes	
Discriminatory practices	Interpersonal discrimination	
	• Stereotyping, myths, and prejudice	
	• Devaluation and Othering	
	• Invalidation and silencing	
	• Withholding information and refusing treatment	
	Institutional discrimination	
	• Interpretation services and time constraints	
	• Room assignment, admissions, and IT system	
	• Lack of competencies among mental health providers, adapted treatment options, and environments	
Strategies and reactions	Mental healthcare service users:	Mental healthcare providers:
	• Confrontation and asking for support	• Trivialization and defensiveness
	• Withdrawal and selective narration	• Acknowledgment and support
Effects of discriminatory practices on service users	• Emotional response	
	• Changes in mental healthcare utilization	
	• Deterioration of mental health status

### Theme 1: Discriminatory Practices

#### Theme 1a: Interpersonal Discrimination

The interviewees described different discriminatory practices on the interpersonal level: stereotyping, myths, and prejudice; devaluation and Othering; silencing and invalidation; and withholding information and refusing treatment.

##### Stereotyping, Myths, and Prejudice

Users and providers suggested that stereotyping, myths, and prejudice negatively influenced communication, diagnoses, and care. Stereotyping and prejudice was prevalent for trans users with mental distress. Matthias (*white* and Russian-German, cis man, mental healthcare provider) described challenges in accessing healthcare settings faced by trans people based on prejudice. He mentioned some colleagues fearing the “social contagion” of trans identities if they allowed trans users to be admitted on their ward. In addition, as Chris (*white*, nonbinary, psychosocial counselor) highlighted, trans people felt pressured to act in accordance with prevalent ideas of how trans people should appear and which body features they should desire, referred to as “trans myths.” Such trans myths reproduced gendered social norms, highlighting the intersection of sexism and trans normativity. For example, Alex (*white*, trans man, service user) narrated how his wish to carry a child and to breastfeed was invalidated by his therapist who expected “authentic” transness to align with a stereotypical ideas of masculinity, and led to the questioning of his trans identity. Johannes (*white*, trans* man, service user) explained that his therapist discouraged him from going on hormonal medication by stressing the beauty of female bodies: “But women’s bodies are much more beautiful. Why do you want to change your body*?*”, thereby reproducing intersecting trans normativity and sexism. Moreover, both Toni (*white*, trans* and nonbinary, service user) and Alex (*white*, trans man, service user) highlighted a specific type of invisibility at the intersection of being trans and being neurodivergent, complicating getting diagnosed with autism and attention deficit hyperactivity disorder, respectively.

Many interviewees also stressed stereotyping based on racialization and racism. Momo (POC, cis woman, service user) recalled stereotyping by her attending senior physician. She quoted him in generalizations such as “Yes, I know, you Turks […], you are all such and such.” Interviewees suggested that racist stereotypes could undermine the appropriate uptake of mental health concerns described by racialized service users. Jan (*white*, cis man, mental healthcare provider) reported how their feelings of sadness and hopelessness were delegitimized as “whining depression.” Racist stereotypes also intersected with sexism: Rebecca (Black, cis woman, mental healthcare provider) explained that in her experience, the diagnosis “trance and possession disorder” was exclusively given to Black people, especially to Black women with symptoms of dissociation in the context of traumatic experiences. Black men, in her experience, were constructed as aggressive or dangerous. To exemplify, Rebecca mentioned the case of a male patient who was falsely treated for trance and possession disorder until he was eventually diagnosed and treated for chronic psychosis. Before receiving suitable treatment, he had been in conflict with the police on multiple occasions.

##### Devaluation and Othering

Stereotypes and myths were often attached to derogatory judgments about racialized non-*white* and positive attribution to *white* service users. Philipp (*white*, cis man, psychosocial counselor) assumed that many therapists unconsciously hold negative views about people of color. Such assumptions became explicit in some of the interviews with *white* mental healthcare providers, for instance, when Julia (*white*, cis woman, mental healthcare provider) commented on racialized male service users: “I’m not xenophobic, but the fact that certain […], we have a lot of foreigners and their attitude of entitlement, that’s where I sometimes reach my limits.”

Furthermore, in conversation with non-*white* service users, psychiatric staff were reported to mention *white* Western norms as the cultural standard, from which non-*white* service users were assumed to differ. Layla (Arab, nonbinary, service user) recalls a psychologist’s comment:

With the first psychologist, the hardest comments that I’ll never forget, like when I said that I wanted to live in freedom […] and then she said: “Yes, like a Western woman, you mean?”

In the therapist’s comment, Layla is not only Othered^5^ based on assumptions about their cultural background but also misgendered, leading to a double misrecognition. Interviewees described various forms of Othering, in which individuals were perceived and treated as a homogeneous group rather than as unique individuals, especially based on racism. Mika (Arab, cis man, service user) reported that during his psychiatric in-patient stay, the ward psychologist told him he could have stayed in his “home country.” Ruben (Latinx and POC, cis man, mental healthcare provider) was asked by a service user to “go back to his home country” and a colleague from another hospital refused to talk to him, instead requesting to speak with a “native colleague,” highlighting that Othering also concerned non-*white* providers. During her admission interview, Momo (POC, cis woman, service user) felt Othered as a woman of color by the senior physician, for instance, when he praised her German (“without any accent”).

##### Invalidations and Silencing

Many participants described situations in which communication was impaired due to silencing, that is, practices by which a person is prevented from speaking or being listened to due to power relations. Silencing was especially noted at the intersection of cis-sexism, racism, and mental illness discrimination. Momo (POC, cis woman, service user), for instance, explained how she was invalidated when she told her therapist about having experienced racist discrimination by the senior physician (“[The therapist] said: ‘He doesn’t mean that in a racist way. He was once in Turkey and he learned to speak Turkish’”). Also, her experiences of everyday racism and its impact on her mental health were invalidated and questioned by her therapist: “Do you really believe that? Do you really think that’s racist? Maybe the person was in a bad mood.” Ultimately, Momo felt that the underlying cause of her depression was unintelligible to the psychiatric staff: “In other words, I had to completely leave out the part that led to depression because I was denied it.” Power imbalances between professionals and service users compounded the invalidation based on gendered racism. Therefore, when experiencing racism from a senior physician, Layla (Arab, nonbinary, service user) felt unable to use the words “discrimination” and “racism” because they feared that this would have harmful effects on the ongoing therapy.

Such power imbalances and hierarchies were also influential in cases of misgendering and deadnaming, prevalent forms of invalidation reported by nonbinary and trans users. Johannes (*white*, trans* man, service user) reported that staff members used the correct pronouns in the first week during his in-patient psychiatric stay but stopped doing so in the second week when the senior physician prohibited the other mental healthcare providers from using the name.

##### Withholding Information and Refusing Treatment

Service users felt that psychiatric staff exercised power by withholding information or treatment in mental health crises. Toni (*white*, trans* and nonbinary, service user), for instance, reported being transferred between different hospitals without being evaluated by a mental healthcare professional during an acute suicidal crisis. Finally, when Toni was assessed by a doctor in the emergency room, the doctor offered them “treatment” for their gender identity rather than addressing the suicidal crisis they were experiencing. Here, the doctor holds particular power to define Toni’s situation based on his position as a professional, as a cis person and as a man. As an asylum seeker, Mika (Arab, cis man, service user) encountered numerous rejections due to uncertainty surrounding his insurance status when seeking emergency admission for acute health crises. After finally being admitted to a psychiatric ward, he recalls not receiving any medical treatment for several days. Furthermore, the hospital staff failed to provide him with any translated information about daily routines, including access to food.

#### Theme 1b: Institutional Discrimination

Interviewees noted unequal treatment due to practices, policies, and procedures in the mental healthcare sector located at an institutional level.

##### Interpretation Services and Time Constraints

A lack of interpretation services was identified as a major barrier to care. Both Philipp (*white*, cis man, psychosocial counselor) and Lou (*white*, no gender category, psychosocial counselor) explained that it is difficult to find adequate treatment for patients who require interpretation services. In their experience, service users were frequently denied admission to psychiatric care due to insufficient language proficiency and a lack of interpretation services. Mental healthcare providers confirmed this impression and explained that people who spoke German with an accent were often rejected by phone admission services, even if appointments were available. In cases where non-German-speaking service users were treated, they were often asked to provide their own translator, for example, through relatives. Rebecca (Black, cis woman, mental healthcare provider) raised different ethical concerns, such as a lack of confidentiality when relatives or cleaning staff were asked to take on the role of interpreters. Additionally, organizational practices in psychiatric hospitals often do not allow additional time for complex communication situations (e.g., communicating with a non-German-speaking service user in acute mental distress). Finally, the psychosocial counselors stressed that professional translators often lack LGBTQIA+ competencies, which compounds communication barriers for non-German speaking LGBTQIA+ service users and particularly fuels the exclusion of LGBTQIA+ service users of color.

##### Room Assignment, Admissions, and IT Systems

Many trans and nonbinary individuals faced issues with inflexible room assignments. Some participants reported that trans individuals were placed in rooms based on their assigned gender at birth or given single rooms, and service users were often not asked about their preferences. Matthias (*white* and Russian-German, cis man, mental healthcare provider) related discriminatory room assignments for trans and nonbinary service users to the lack of institutional guidelines on gender diversity. In some cases, the inability to choose suitable room arrangements led to the early termination of treatment, as described by Leonie (*white*, trans woman, service user). Institutional discrimination may also operate through the IT systems, as Matthias (*white* and Russian-German, cis man, mental healthcare provider) noted. Difficulties in entering correct names and pronouns for service users in the IT system may lead to recurring instances of misgendering each time a user is in contact with new staff.

##### Lack of Competencies Among Mental Health Providers, Adapted Treatment Options, and Environments

Psychiatric staff were described as lacking knowledge about and skills regarding structural discrimination, especially around racism and LGBTQIA+ discrimination. Rebecca (Black, cis woman, mental healthcare provider) explained that many psychotherapists are unaware that existing diagnostic tools allow mental healthcare professionals to record experiences of discrimination and their impact on patients’ mental health. She further noted the lack of German-language resources and manuals to address racism in psychotherapy: “So there is no racism-sensitive therapy or psychotherapy here in Germany. It just doesn’t exist.”

Several participants identified the lack of competencies as an institutional problem that may lead to treatment refusals on an individual level, especially if providers do not feel qualified to provide suitable care. Lack of competencies particularly concerns service users affected by multiple forms of discrimination, for example, LGBTQIA+ racialized service users. Moreover, interviewees stressed that if specific topics are not included in the formal training (e.g., the interrelationship of racism, LGBTQIA+ discrimination, and mental health), psychiatric staff may assume that these topics lie beyond their area of responsibility. This, again, particularly affects service users who experience discrimination based on multiple systems of oppression. They remain unseen and misunderstood with regard to multiple aspects of their identity. Rebecca (Black, cis woman, mental healthcare provider) pointed out that many people who need mental healthcare cannot access it, particularly those seeking racism-sensitive treatment due to institutional setup and treatment options:

So the most important discrimination I notice is that people don’t even arrive at all. You can see it, but maybe you can’t see it. I’ve always been surprised by the fact that certain people arrive in psychiatric wards and others just don’t.

Care environments may also be perceived as hostile or exclusionary. The psychiatric clinic where Alex (*white*, trans* man, service user) received treatment displayed posters and leaflets with homophobic and transphobic messaging.

### Theme 2: Strategies and Reactions

Service users and psychiatric staff employ various strategies to navigate services and show different reactions when faced with discriminatory practices. The following subthemes were identified among service users: confrontation, asking for support, withdrawal, and selective narration. Psychiatric staff most commonly either trivialized and reacted defensively to discrimination or recognized its presence.

#### Theme 2a: Service Users’ Strategies and Reactions

##### Confrontation and Asking for Support

In some cases, users confronted physicians directly when they experienced or observed discrimination. Matthias (*white* and Russian-German, cis man, mental healthcare provider) recalled a young trans service user challenging a senior physician who had repeatedly discriminated against them:

The patient was incredibly overwhelmed by the situation and then, I think, reacted quite well and said that she didn’t want to talk to him, to a white cis man, but that it was so intimate for her that she would like to discuss it with a […], with a trans person, which I thought was a really good […], really good response at that moment, which, of course, totally shocked the senior physician.

Similarly, Layla (Arab, nonbinary, service user) confronted psychiatric staff who refused to respect their wish to be addressed by their first and last name (rather than being called Mrs.), and Momo (POC, cis woman, service user) asked her psychotherapist for support after having been discriminated against by the attending physician. Both Momo and Layla stressed the particular burden of challenging discrimination from positions of multiple disadvantages, based on gender discrimination, racism, and current mental distress. These incidents occurred at the beginning of each in-patient stay, indicating that service users first try to adapt the structures to their own needs.

##### Withdrawal and Selective Narration

When their efforts to adapt their environment failed, service users employed withdrawal and selective narration as strategies to avoid further negative experiences in mental healthcare. Users explained that they had self-censored their testimonies in order to benefit as much as possible from therapy, in cases where they experienced their environment to lack the necessary know-how to understand them. Service users described quickly recognizing when specific topics were better not addressed directly to avoid possible discrimination, even if this undermines potential therapy success, based on past negative experiences. Momo experienced that her intersectional social identity was unintelligible on multiple respects, so that she decided to self-censor her narrative:

If you leave out such an important part, then the therapy wasn’t as successful, because I also have post-traumatic stress disorder and a lot of it comes from my childhood […] but also experiences of racism and also a refugee background, so that means I couldn’t talk about these things either in the group nor in the individual therapy, because no understanding or my appearance didn’t fit into their picture, so I’m a lesbian, an atheist and I must express myself really brilliantly for them, so I can’t have any problems in Germany.

Toni (*white*, trans* and nonbinary, service user) explained that if they only ever talk about being trans at the end of a medical encounter, the risk of negative bias on the part of the healthcare provider is minimized. Several participants also reported feeling more confident in their therapy setting when they anticipated discrimination to occur, because this made them feel more resilient.

#### Theme 2b: Mental Healthcare Providers’ Strategies and Reactions

##### Trivialization and Defensiveness

Mental healthcare providers showed different responses when they were confronted with discriminatory practices. Ruben (Latinx and POC, cis man, mental healthcare provider) recalled that when he told his coworkers about an instance of racist discrimination by a colleague, they minimized his experience: “White Germans somehow don’t take racism seriously enough, [they say: ‘there is] no racism here in Germany and it’s just a small thing”*.* During one interview, Jana (*white*, cis woman, mental healthcare provider) compared correcting misgendering of a trans person to making someone aware of a food allergy, thereby trivializing the psychological burden of trans-negativity. Service users also described psychiatric staff becoming defensive when confronted about their discriminatory practices. Some staff used gendered and racialized mental illness stereotypes to invalidate such criticisms. Layla (Arab, nonbinary, service user) recalled being called “overly sensitive” due to their mental illness when they criticized a psychiatrist of reproducing racist stereotypes about refugees in Germany.

Finally, mental healthcare providers also attempted to justify their inadequate treatment options for individuals with diverse gender identities or non-German speakers by claiming that this only affected a small number of people, or that preparing more suitable therapy materials would be too time-consuming.

##### Acknowledgment and Support

Several of the healthcare providers interviewed recognized discriminatory practices within mental healthcare, especially the burden of lacking competencies around racism, lack of interpretation services, and lack of structures prepared for trans and nonbinary service users. Several participants expressed shock and anger about blatant discriminatory behavior on the part of colleagues. Matthias (*white* and Russian-German, cis man, mental healthcare provider) explained:

I actually had the feeling in […], in the year 2022, as if I was sitting in a psychiatric ward before 1980 and a homosexual patient was sitting in front of me, and that’s exactly how it felt, and I say that now as a gay man myself, because I thought to myself, that could really have been forty years ago, in the psychiatric ward, where somewhere an old white man is just throwing around some supposed technical terms to pathologize me.

As a consequence, he decided to support trans service users the best he could within inflexible structures and static hierarchies, for instance, by informing them about the discriminatory treatment that they will potentially experience:

So I actually tell almost all patients at the beginning that there is a possibility that they will experience discrimination here in the clinic because of their gender identity and that I am very sorry in advance and that it is currently a battle that we are fighting here to some extent.

Some mental health providers acknowledged that they lacked the necessary competencies to respond appropriately to instances of reported racism or the impact of racism on service users’ mental health, and acknowledged that their own judgments were sometimes influenced by negative stereotypes.

### Theme 3: Effects of Discriminatory Practices on Service Users

The participants described the following main effects of the experience of discriminatory practices on marginalized mental healthcare service users: emotional response, changes in mental healthcare use, and worsening of mental health.

#### Emotional Response

Service users reported intense emotions in response to experiencing discriminatory practices in mental healthcare. They described feelings of anger, fear, helplessness, loneliness, self-doubt, and distrust of those providing treatment. Momo (POC, cis woman, service user), for instance, expressed her profound shock and frustration after being confronted with intersectional heterosexism and racism within mental healthcare. Alex (*white*, trans* man, service user) described that discriminatory treatment triggered extreme fears of being dismissed from the in-patient service. Additionally, some service users described feeling angry when providers focused on their social identity, for example, as a trans person, instead on their current mental health needs.

#### Changes in Mental Healthcare Utilization

Different participants explained that marginalized service users may react to discriminatory practices by being more reluctant to use mental healthcare services. Layla (Arab, nonbinary, service user) stated that if they had another acute mental health crisis, they would prefer not to seek therapy at all, based on their previous negative experiences of intersecting discrimination within mental healthcare. In line with this, mental healthcare providers noted that they experienced marginalized service users to be more likely to be admitted in acute mental health crises. Additionally, Rebecca (Black, cis woman, mental healthcare provider) believed that marginalized service users, especially those who are racialized and LGBTQIA+, spent a large amount of time looking for providers with adequate competencies to address discrimination.

#### Deterioration of Mental Health Status

Providers stressed that experiencing discrimination, especially within mental healthcare, can worsen users’ mental health status and lead to trauma-related disorders or retraumatization. Additionally, as Peter (*white*, cis man, mental healthcare provider) explains, having to hide one’s emotions and avoid certain content can lead to various mental health difficulties and mental health crises:

Yes, I think that if someone has the experience: “I have to keep quiet about what actually moves me and how I experience myself as a person,” that can lead to a whole series of psychological difficulties, starting with “I generally can’t trust others,” a general mistrust, “I can’t”—let’s say—“open up emotionally” and so on, right through to questions of sexuality and so on. So I think there are lots and lots of things that play a role here, right up to suicidal crises.

Alex (*white*, trans* man, service user) stated that the persistent discrimination he faced during his in-patient stay, based on his gender identity, led to the development of an anxiety disorder. Leonie (*white*, trans woman, service user) explained that being forced to sleep in a men’s room hindered her recovery process. Several service users thought that they required consecutive psychotherapy to cope with the experiences of discrimination in mental healthcare. This was supported by Rebecca (Black, cis woman, mental healthcare provider) who stated that she regularly has to address instances of discrimination that her clients have experienced with other therapists.

## Discussion

This study examined discriminatory practices within mental healthcare through an intersectional lens, highlighting their manifestation on both interpersonal and institutional levels. Service users and mental healthcare providers developed various strategies to navigate the different forms of discrimination. We discuss the results under consideration of two major systems of oppression identified across the interviews in their intersections with mental illness: cis-heteronormativity and Whiteness.

### Whiteness, Cis-heteronormativity, and Mental Illness

Many of the discriminatory practices identified in our study can be related to the effects of structural cis-heteronormativity and institutional Whiteness. Cis-heteronormativity refers to the normalizing of a gender identity aligning with the sex ascribed at birth (cis-normativity) and the exclusive sexual or romantic attraction to the “opposite” gender (heterosexuality), with the simultaneous pathologizing of LGBTQIA+ lives (Hutfless, 2019; Nieder et al., 2020). Cis-heteronormativity can be understood as a structural feature of psychiatry so far as it is rooted in a lack of attention to the needs of LGBTQIA+ people in research, education, and practice. Whiteness describes the simultaneous positioning of *white* norms and epistemologies as the standard, and the devaluing of non-*white* approaches, while this process itself is rendered invisible.^6^ Whiteness has deep historical roots in colonialism and is built into psychiatric institutions, for instance, taking form as diagnostic categories (Lazaridou & Fernando, 2022; Lerch, 2019; Yeboah et al., 2024).

One main concern that reduces the access to and quality of care for marginalized service users is healthcare providers’ lack of knowledge regarding structural discrimination. Two systematic reviews identified a limited knowledge of LGBTQIA+ individuals’ needs and stigmatization in healthcare as major barriers to healthcare (Snow et al., 2019; Rees et al., 2021). Regarding structural racism, research shows that its cumulative effects may lead to trauma, chronic stress, and increased prevalence of mental illness. Having competencies on structural racism is, therefore, highly relevant for the care of racialized users (DeZIM, 2023; Patil et al., 2018). However, our results, aligning with further research, indicate that mental healthcare staff lack competencies to integrate the influence of intersectional racism in their therapeutic approach (Aikins et al., 2021; Hamed et al., 2022; Hempeler et al., 2024; Yeboah et al., 2024). Our results suggest that this also applies to providers who acknowledge the impact of structural discrimination, highlighting the necessity to systemically embed such competencies in healthcare provider’s education and training (Lazaridou & Fernando, 2022; Metzl & Hansen, 2018).

Our study demonstrated how mental healthcare providers, as a consequence of lacking robust knowledge regarding gender diversity, relied on stereotypical assumptions and myths about gender diversity, a concern also raised in the literature (Snow et al., 2019; Velasco et al., 2022). Such stereotypes are shaped by societal assumptions and legal regulations on a structural level. Difficulties in imagining a pregnant trans man, for example, may be informed by the requirement of sterilization for the change of one’s legal gender in official documents, in place in Germany until 2011 (BVerfG, 2011). Similarly, assumptions about the “correct” way of being transgender, based on a binary understanding of gender (Lampe, 2024), may be strengthened by healthcare insurance requirements for covering treatment costs, which in Germany exclude nonbinary people from gender affirmative healthcare (BSG, 2023).

Our study also highlighted intersectional gendered and racialized stereotypes (Hempeler et al., 2024; McMaster et al., 2021). Further research suggests that pejorative stereotypes about Black women in psychotherapy may influence the interpretation of symptoms presented during mental distress and thereby impact the quality of care (Ashley, 2014). Furthermore, the stereotypical construction of Black male service users as “the mad, bad, and [dangerous]” (Keating, 2016, p. 174) may be associated with the heightened use of coercive measures in Black service users (Faissner & Braun, 2024), documenting the harmful effects of stereotyping.

Our results also demonstrate how institutional level practices may translate into interpersonal forms of discrimination. For instance, binary documentation systems or the lack of guidelines for room assignment may reproduce institutional cis-heteronormativity (Kahl et al., 2022), and may result in misgendering. In our as well as other qualitative studies, participants experienced misgendering as extremely harmful (Brokmeier et al., 2022; Hempeler et al., 2024; McCullough et al., 2017). Given the high mental health burden of LGBTQIA+ healthcare users based on minority stress (Hoy-Ellis, 2023), LGBTQIA+-friendly services, affirmative care, and visible acceptance and support are vital (Hempeler et al., 2024; Rees et al., 2021).

Additionally, the systematic lack of translation services, highlighted in our results, can be interpreted as one feature of institutional Whiteness (Aikins et al., 2021; DeZIM, 2023; Schödwell et al., 2022). Based on their results from the Afrozensus, Aikins et al. (2021) argue that insufficient translation services can result in a lack of appropriate information for users, undermine the informed consent standard, and lead to treatment exclusions. Schödwell et al. (2022) stress that especially economic constraints, such as the lack of funding for translation services, support language-based exclusions. LGBTQIA+ service users who require interpretation services may face additional challenges because not all interpreters have sufficient LGBTQIA+ competencies. This particularly concerns LGBTQIA+ refugees, a group documented as vulnerable to experiencing discrimination and poor mental health in Germany (Golembe et al., 2021).

Our study and the NaDiRa results both indicate that marginalized service users encounter multiple access barriers to mental healthcare (DeZIM, 2023). The NaDiRa shows that service users vulnerable to racism are more likely to abandon looking for psychotherapy: While around one in five (21%) *white* people had given up looking for therapy, one in three (35%) non-*white* people had done so (*n* = 2120) (DeZIM, 2023). These findings support the influence of discrimination and access barriers on decreased service utilization assumed in the literature (DeZIM, 2023; Hamed et al., 2022; Henderson et al., 2015).

Finally, both institutional Whiteness and cis-heteronormativity may manifest in the systematic impossibility of addressing and criticizing its effects. According to a scoping review on racism in healthcare, providers tend to perceive healthcare as impartial and, thus, stay oblivious to discriminatory practices within healthcare (Hamed et al., 2022), a dynamic illustrated in the defensive and trivializing reactions among mental healthcare providers in our study.

### Intersectionality: Unintelligibility, Silencing, and Othering

Our study demonstrates how intersecting systems of discrimination create particular challenges for marginalized service users and mental healthcare providers. Three interrelated processes that impede healthcare provision for marginalized service users emerged as particularly important: unintelligibility, silencing, and Othering. We suggest that these are best understood in intersectional terms, noting the simultaneity and complexity of marginalization processes (Carastathis, 2014).

Mental healthcare providers appeared unable to grasp the complexity of the lived realities and identities of service users at the intersections of multiple systems of discrimination. Such an inability on behalf of providers can be termed “intersectional insensitivity” (Hempeler et al., 2024; McCullough et al., 2017). The compounded disadvantage becomes clear in Momo’s account. Her identity as a lesbian woman academic with a Turkish background was not conceivable within stereotypical expectations. Because the providers imagined Turkish cis-women as oppressed, heterosexual, and illiterate, and lesbian women as emancipated and *white*, Momo interrupted their narrow framework of intelligibility. As a consequence, Momo was left misunderstood and felt compelled to leave out essential aspects of her identity and experience. This can be understood as a form of *silencing*, which refers to processes by which members of marginalized groups are prevented from speaking out or being adequately heard due to power structures (Dotson, 2011). In mental healthcare, silencing may occur when staff discards people’s complaints about racism, transphobia, heterosexism, and their intersections, or invalidates their suffering (Aikins et al., 2021; Hamad et al., 2020; Hempeler et al., 2024). A specific form of silencing, “smothering,” occurs if a speaker chooses to self-censor parts of what they would like to communicate to protect themselves from adverse reactions from an audience that is unfamiliar with the relevant knowledge (Dotson, 2011). While other authors have emphasized its emotional burden (DeZIM, 2023), in our study, “selective narration” was also presented as a protective strategy of marginalized service users, which made them feel self-effective within a discriminatory environment. Intersectional stereotyping and invalidations led to Othering, a dynamic also described in the NaDiRa (DeZIM, 2023). In line with our results, Bhugra et al. (2023) highlight the impact of Othering in mental healthcare on therapeutic outcomes, healthcare use, and service users’ well-being.

Our results support that intersecting systems of oppression undermine accessible, acceptable, and supportive mental healthcare for marginalized social groups via discriminatory practices resulting in unintelligibility, silencing and Othering. Marginalized service users, in turn, may respond with emotional distress, decreased trust in mental healthcare institutions and reduced service use (DeZIM, 2023; Hamed et al., 2020, 2022; Lerch, 2019; Progovac et al., 2020), a dynamic that can exacerbate intersectional health inequalities (Fagrell Trygg et al., 2019).

### Limitations

Some limitations must be acknowledged in interpreting the results of this study. First, we acknowledge that the selection of participants may have involved a selection bias. The leaflets informed that the study analyzed experiences of discrimination in mental healthcare. Thus, service users who have specific knowledge of the topic may have been more likely to participate in the study. However, the dynamics described by the service users affected were confirmed by both socially marginalized and non-marginalized mental healthcare providers, as well as the psychosocial counselors; therefore, we assume our results to be reliable.

Second, we were unable to recruit Black service users, and overall, more *white* people compared to non-*white* people participated in the study. This reluctance could be attributed to the fact that the study was conducted within *white* academic structures. More community-based research projects on intersectionality and racism within German mental healthcare would be beneficial to address this. Additionally, social class appears to be an important access barrier to mental healthcare (Barnett et al., 2023) and should be analyzed as it intersects with racism and cis-heteronormativity.

Finally, in line with an intersectional research practice, we aimed to involve study participants through participatory elements, such as participatory data analysis sessions and member checking. Participation in data analysis sessions decreased over time, and some participants declined participation in the member checking altogether, which indicates that the research design might have included too many different formats that exceeded many interviewees’ resources and availability. However, the collaborative study team with coresearchers from anti-discrimination organizations proved to be a constructive and successful format for joint study coordination.

## Conclusion

This study is, to the best of our knowledge, the first to investigate discriminatory practices within mental healthcare services from the perspective of mental healthcare service users, providers, and psychosocial counselors in Germany using an intersectional approach. Addressing discrimination within mental healthcare is an urgent task for healthcare providers, policymakers, and institutions. Future research must focus on developing and evaluating interventions that can effectively mitigate the harmful effects of discrimination in mental healthcare.

## Supplemental Material

Supplemental Material - Discriminatory Practices in the German Mental Healthcare System: An Intersectional Grounded Theory Study Supplemental Material for Discriminatory Practices in the German Mental Healthcare System: An Intersectional Grounded Theory Study by Neneh Rosalía Quadflieg, Patiani Batchati, Alva Träbert, Eike Leidgens, Georg Juckel, Jakov Gather, Amma Yeboah, and Mirjam Faissner in Qualitative Health Research

## Notes

1. Marginalized groups are population groups with limited access to resources, opportunities, and rights compared to privileged groups.

2. With the capitalization of “Black,” we stress its social construction, in line with the preferences of community members, journalists, academics, and activists. We lowercase and italicize *white* to highlight that Whiteness is also constructed and to prevent Whiteness from being seen as an unquestioned standard or norm.

3. Forms and effects of structural discrimination faced by people with mental illness are documented in the literature focusing on mental illness stigma (e.g., Thornicroft et al., 2022). While our research is compatible with stigma research that considers the socio-structural aspects of power relations (Link & Phelan, 2001), we used intersectionality as the framework to start from the experiences of multiple discrimination.

4. This focus emerged in the course of collaboration within the study team based on the experiences of the anti-discrimination counselors.

5. *Othering* was conceptualized by postcolonial theorists (e.g., Said, 2021; Spivak, 1988) and refers to the process of constructing a group as homogenous, inherently different, and inferior compared to an in-group.

6. Fanon (2019/1986) and Mills (2008) developed important analyses of Whiteness.
