# Supplementary material for: Discriminatory Practices in the German Mental Healthcare System: An Intersectional Grounded Theory Study
Source: Qual Health Res. 2025 May 15;36(9):1027–42. doi: 10.1177/10497323251325793 (PMC13338484; doi:10.1177/10497323251325793)
Supplement: Supplemental Material - Discriminatory Practices in the German Mental Healthcare System: An Intersectional Grounded Theory Study [file sj-pdf-1-qhr-10.1177_10497323251325793.pdf]

Discriminatory Practices in the German Mental Healthcare System – an Intersectional Grounded Theory Study

Online Supplement

Supplement A: Interview Guides

**Guiding questions**	**Further questions for clarification**
**Narrative prompt: Counseling strategies**<br><br>1) First of all, thank you for taking the time for this interview. You work in psychosocial counseling for [insert social group here].<br><br>What does typical counseling look like for you for people who experience discrimination in mental healthcare?<br><br>2) What strategies for dealing with experiences of discrimination have been reported to you by those affected?	
**Precision questions: Intersectional discrimination in mental healthcare**<br><br>3) Let us now come to the topic of discrimination in more detail. From your point of view, what is discrimination in mental healthcare like?<br><br>4) What connection do you see between experiences of discrimination, mental health and access to mental healthcare?<br><br>5) In order to make different experiences of discrimination visible, the concept of intersectionality is sometimes used. In your experience, how is discrimination based on mental illness related to other systems of discrimination (such as racism, heterosexism, ableism)? What relevance does the concept of intersectionality have here?<br><br>6) What effects can intersectional discrimination in mental healthcare have on those affected?	Can you give examples on an individual, institutional and structural level?<br><br>Sometimes people also talk about intersectional stigma. Can you say something about this?<br><br>In your experience, how much space is given to the topic of discrimination in psychosocial counseling?<br><br>What effects do you observe at a medical, social, and societal level for those affected?<br><br>What effects do you observe on the behavior and self-image of those affected?
**Evaluation questions: Interview guide for service users and mental healthcare providers**<br><br>We have sent you in advance an interview guide on experiences of discrimination in mental healthcare for service users and mental healthcare providers.<br><br>7) What is your general evaluation of the guidelines? Do you have any suggestions for improvements? Would you ask individual questions differently? In your opinion, are all the important questions covered?	

**Closing questions**<br><br>8) Is there anything else you would like to mention?<br><br>9) How was the interview for you?	

Table 1 *Interview guide psychosocial counselors*

**Guiding questions**	**Further questions for clarification**
**Opening questions**<br><br>1) You have already had contact with mental healthcare services. Tell us how this came about.<br><br>2) How did the consultations go? Can you tell us about positive and negative experiences in contact with mental healthcare? Please name several experiences if you wish.<br><br>3) Mental healthcare can involve the use of coercion, such as involuntary treatment, restraint, or isolation. Are there situations during treatment in which you have experienced coercion? If so, what were these situations? Please describe them.	Named people and their role<br><br>Quality of relationships<br><br>Places mentioned, healthcare spaces<br><br>Experience(s) of discrimination before treatment?<br><br>Process or sudden/unexpected event?<br><br>Admission associated with coercion?
**Questions for reflection**<br><br>**Discrimination**<br><br>Discrimination can take very different forms. In some situations, it manifests in a direct way, while discrimination is sometimes hidden. It is also possible that you did not perceive it as discrimination.<br><br>4) From today's perspective, how would you evaluate the situations described? Are there situations in which you experienced discrimination in mental healthcare? If so, what were these situations? Please tell us about one situation.<br><br>5) How did you respond to instances of discrimination? How were you able to accept mental healthcare treatment despite the experiences of discrimination? Was there anything that helped you?<br><br>6) How do you think discrimination could have been avoided in the situations described?<br><br>**Coercion**<br><br>7) Do you see any links between your experiences of discrimination and the experience of coercion?<br><br>8) In the situations in which discrimination was associated with coercion or pressure, what do you think could have prevented you from feeling coerced in these situations?	To give more explanations about discrimination:<br><br>Have there been situations in which you did not receive the necessary treatment because you had the feeling that the person responsible was prejudiced against you?<br><br>To what do you attribute the discrimination?<br><br>Various intersectional forms of discrimination mentioned:<br><br>Racism / sexism / ableism / classism / heteronormativism / other forms of discrimination?<br><br>Inter-individual / institutional / structural level addressed?<br><br>Connection between discrimination and stigmatization due to mental illness?<br><br>Own change in behavior due to your experience of discrimination?<br><br>Own emotional feelings?<br><br>Connection to forms of treatment/pressure/coercion<br><br>Coping strategies?

**Effects**<br><br>9) From today's perspective, how do you think the experience of discrimination in clinic XY (example) has affected your further life/well-being? Is the experience you described still important and present today? If so, in which situations?	Evaluation of discrimination?
**Evaluation questions**<br><br>**10)** How significant do you consider the problem of discrimination in mental healthcare to be?<br><br>**11)** If you, as a person affected, had the opportunity to help design the treatment of people with mental illness, what are your suggestions for improvement?	Significance of the problem?<br><br>Concrete measures?<br><br>Wishes?
**Closing questions**<br><br>**12)** Have we forgotten anything you would like to mention?<br><br>**13)** We are now at the end of our conversation. How do you feel? Do you have any questions?	Evaluation of the possibility of change<br><br>Future prospects with own design possible?

Table 2 *Interview guide service users*

**Guiding questions**	**Further questions for clarification**
**Opening questions:**<br><br>**1)** Thank you for taking the time to do this interview. You work in mental healthcare as a [insert role]. Please tell us about a typical working day. What are your tasks or duties?<br><br>**2**) Discrimination may occur in mental healthcare. It can take many different forms. Sometimes, it is very direct and sometimes more hidden. In addition, discriminatory behavior can happen "by accident" and is not always deliberate or intentional. With this in mind, please tell us about a situation that you have experienced as discriminatory. How did this situation come about and what exactly happened?<br><br>**3)** Can you remember situations in which the quality of treatment of patients was compromised due to discrimination? If yes, which situations were these? Please describe them.<br><br>**4**) Coercion may be used in mental healthcare. Do you remember situations in which discrimination was associated with (psychological) pressure or coercion (compulsory treatment, restraint or isolation)? If yes, which situations were these? Please describe them.	Named places, healthcare spaces, activities<br><br>Discrimination as a process or sudden/unexpected event?<br><br>Own experience of discrimination, observed discrimination?<br><br>Connection to forms of treatment/pressure/coercion
**Questions for reflection**	

5) In these situations that you perceived as discriminatory, to what would you attribute the discrimination?<br><br>6) What do you think are particular aspects of discrimination specific to mental healthcare?<br><br>**7**) What effects do you think discrimination has on mental healthcare and the mental health of those affected?	Different intersectional forms of discrimination mentioned:<br><br>racism / sexism / ableism / classism / heteronormativism / other forms of discrimination?<br><br>Inter-individual / institutional / structural level addressed?<br><br>Connection between discrimination and stigmatization due to mental illness?
**Evaluation questions**<br><br>8) What kind of awareness of discrimination do you think there is among mental healthcare professionals?<br><br>9) In situations where you have discriminated against or observed discrimination, how do you think discrimination could have been prevented? How would you wish to act in a similar situation in the future? What would anti-discriminatory mental healthcare have looked like?<br><br>10) Where discrimination was associated with coercion or pressure, what do you think could have prevented discriminated people from feeling coercion or pressure in these situations?<br><br>11) If you have experienced discrimination yourself, in situations where you experienced discrimination, how were you able to carry out your professional activities? Was there anything that helped you? What do you wish for the future?	Coping strategies?<br><br>Attitudes towards discrimination?<br><br>Relationship to formal and informal coercion?<br><br>Assessment of the possibilities for change.
**Closing questions**<br><br>**12)** Have we forgotten anything you would like to mention?<br><br>**13)** How was the interview for you?	

Table 3 *Interview guide mental healthcare providers*

Suppement B: Themes and exemplary quotes

Table 2 Quotes

**MAIN THEMES**	**SUBTHEMES**	**CATEGORIES**	**DEFINITION**	**EXEMPLARY QUOTES FROM RAW DATA**
**1. Discriminatory Practices**	**a) Interpersonal Discrimination**	Stereotyping, myths and prejudice	Stereotyping (association of social group member with specific traits), prejudice (preconceived negative stereotypes), or myths (erroneous narratives) influence interactions, communication, diagnosis, or care in mental healthcare.	**SERVICE USERS**<br>**Toni** (*white*, trans* and nonbinary, service user) on challenges in the interaction between being trans* and being diagnosed with autism: "When I explained that I had autism and [talked about] my childhood experiences, that I was already totally abnormal back then, it was actually completely dismissed. Whether there was an overarching […], that is, a direct connection to being trans, I don't really believe […], I don't think that this was doubted. But I can imagine that it was in the back of their mind."<br><br>**Alex** (*white*, trans man, service user) on challenges in the interaction between being trans and being diagnosed with Attention Deficit Hyperactivity Disorder: "And I had done a bit of reading, so I knew the diagnostic criteria according to ICD and DSM. I also looked at some of the interviews and tests that are done. But they did a lot of other things with me, and then they asked an endless number of questions again and again. It was always about 'trans,' and I honestly don't know what the whole context of the matter was. To be honest, I'm not sure why it took so long. It seems like they just didn't know what to do with me, and I guess I don't fit their strange stereotypes."<br>**Momo** (POC, cis woman, service user) describing how psychiatric staff expressed the assumption that she must have suffered sexist oppression qua being perceived as a Muslim woman: "Yes, that definitely goes together, because as […], well, in their world I was a Muslim woman and as a woman I experienced discrimination, i.e. oppression at the hands of men. [They expected me to be] afraid of men and [that I] can't assert myself. It was

				more this kind of discrimination.”<br><br>**MENTAL HEALTHCARE PROVIDERS**<br><br>**Jan** (*white*, cis man, mental healthcare provider) when asked whether he has noted any prejudices in diagnosing nonbinary and trans patients with personality disorders: “I think so. So it’s actually always prejudiced. So I, I […], it’s in my mind, and I think probably in the minds of most other doctors too, that the first thing you ask yourself [when you see a nonbinary or trans patient] is whether this is also an emotionally unstable personality […] or the first thing you ask yourself is whether there could be an emotionally unstable personality disorder.<br><br>**Rebecca** (Black, cis-woman, mental healthcare provider) on racism and diagnostic processes: “[…] and what I also think is that sometimes, because of language barriers, perhaps because of the picture, but not necessarily the clinical picture, diagnoses are given that are given more frequently in certain groups than in others. So I’m […], I’m always surprised about who is considered to have a psychotic episode, for example, or who is already diagnosed with schizophrenia, where other people with the same symptoms […], so it goes a bit into the […], into the cultural trap of quickly assigning things, or who then […] what this trance and obsession disorder […], well, where I always think, well […], I’m always a bit surprised that this is given and not another dissoci […], so another disorder from this dissociative area, for example. But if you’re – I don’t know – of African origin, then it’s often trance and obsession, when I think like that, yes, I don’t know, I often have problems with it.”<br><br>**PSYCHOSOCIAL COUNSELORS**<br><br>**Chris** (*white*, nonbinary, psychosocial counselor) on prejudices in the treatment of trans and inter patients: “So sometimes there are just wrong treatments or all these things that you know from discrimination or from trans-inter people, that the trans issue is often focused on in the treatment, although someone comes in for something completely different and actually has no question about trans, and the health problems that someone comes in with have nothing to do with the respective treatments. Nevertheless, the

				practitioners then focus very strongly on the trans issue, either with inappropriate curiosity or by constructing connections between, for example, hormone treatment and symptoms that have nothing whatsoever to do with hormone treatment.”
		Devaluation and Othering	Interviewees describe discursive forms of exclusion, devaluation, or homogenizing, based on perceived social groups membership.	**SERVICE USERS**<br>**Momo** (POC, cis woman, service user): “It actually started on my first day. It was a bit of a strange situation. [...] It was the senior doctor, the psychiatrist, and I had to go to him for rounds, so to speak. He first praised me for speaking German without an accent. I said: ‘No, you don’t have to praise me for that. That is really not necessary,’ I told him. Then I asked if he asks all his patients this question and why he asked me.”<br>**Layla** (Arab, nonbinary, service user): “The nurses also called me ‘the foreigner,’ for example. I heard that several times. Also, when I arrived, that was my first experience of that kind. I came into my room, and the woman [with whom I was supposed to share the room] said ‘Hi [...].’ After an hour, though, I saw that she was packing her things and leaving. Turns out she didn’t really want to be in a room with a ‘foreigner.’ The [staff] offered her another room because she wanted to go to the other room.”<br>**Mika** (Arab, cis man, service user): “There was also a manager for the nurses in [German city F], who was actually one of them and a psychologist. I had a quick chat with her.”<br>*Interpreter*: “She then asked me: ‘So, why did you come to us here? Can you give me a quick summary of why you came to us here?’ I also gave her a quick summary, like I did before. […] Then I was told: ‘Unfortunately, that’s your problem.’ [They told me]: ‘This is my country, but not yours […] ‘If you’d stayed in your country, it would never have happened to you.’”<br>**MENTAL HEALTHCARE PROVIDERS**<br>**Ruben** (Latinx and POC, cis man, mental healthcare provider): “Yes, I’ve already had a few instances where racism came up. That’s right. The last one

				was actually here in [hospital C] in [major German city] in the outpatient clinic, because I've been here for a year. […] The last thing here was a patient who didn't get what he wanted from me and then he kind of told me to go back to my country. That's right. That's exactly what happened here."
		Invalidation and silencing	Interviewees describe how information, experiences of discrimination, or aspects of their identity they find important are not heard, believed, or taken seriously, in the context of power structures.	**SERVICE USERS**<br><br>**Layla** (Arab, nonbinary, service user) to the question whether they ever felt discriminated against: "I don't use the words 'discrimination' or 'racism' any more, because I know they're loaded terms. I'm not afraid to use them, but I do so with caution because I know the [mental healthcare provider] is stronger. That's why I wouldn't say that [it was racism]."<br><br>**Momo (**POC, cis woman, service user): "and then I went to my therapist and talked to her about it. [I said]: 'Okay, that really upsets me, I'm also working in the field of anti-racism. That's my job and right now, psychologically, it'', haunting me here' and I told her about it, but she didn't understand. She said: 'The [senior physician] doesn't mean that in a racist way. He used to be in Turkey and he learned to speak Turkish.'"<br><br>**Johannes** (*white*, trans* man, service user) on being misgendered: "The first two stays were in the same clinic and there the thing was that they didn't address me with my desired gender and name, and that even though they knew that the crisis that led me to them, outing with my parents, was just due to this. […] The senior physician said [the staff] couldn't do it and weren't even allowed to do it [address me with my desired gender and name]."<br><br>**MENTAL HEATLTHCARE PROVIDERS**<br><br>**Ruben** (Latinx and POC, cis man, mental healthcare provider) highlighted that ignoring racialized service users another way of invalidation: "And also when you don't understand the patients because they don't speak German well. Then you often simply ignore them a bit, or you don't even try to get in touch with them, because they don't understand anyway and then you don't make any effort. For me, that's also a kind of – yes – discrimination."

		Withholding information refusing treatment	Healthcare providers or institutions do not provide treatment or information (e.g. on treatment options or procedures) to service users in need of treatment or information.	**SERVICE USERS**<br>**Toni** (*white*, trans* and nonbinary, service user) on an experience of treatment refusal: “I was sent from hospital to hospital after a suicide attempt. The hospitals said they weren’t responsible. After four hours, we found a hospital that would let us into the waiting area. We waited there for six hours, and the doctor in charge was very strange. After I had I told her what the situation was, she told me the ‘trans problem’ could be cured and that I could go to therapy for it.”<br>**Mika (**Arab, cis man, service user) on his treatment experience in an inpatient psychiatric ward: “The whole ward, the whole day [long], my […], neighbor, always goes downstairs or upstairs for the therapy plans, but I always stay alone in the ward and I’m not registered in therapy plans or anything. […]”<br>*Interpreter*: “And I went four days without food, without drink and without treatment because […] everything was closed and I couldn’t leave the room. And during the ward round, I also spoke to the doctor about the fact that I hadn’t been given anything, hadn’t eaten anything either, and I spoke to the other nurses and they said: ‘The food is in the kitchen, not here,’ so I had to go there to get it or eat there, even though I couldn’t or didn’t know about it.”<br>**Mika:** “[I didn’t know] that I always have to collect it myself […] Nobody told me anything. Nobody.”<br>**Layla (**Arab, nonbinary, service user): “I stayed hospitalized there for two weeks and most of the time in the room, so ten days, and I still didn’t know until then that I was allowed to go out, for example, even into the garden, which was in front of the hospital. I didn’t know. Nobody told me anything, neither about possibilities of treatment nor about, about the rules that had to be observed there, nothing.”<br>**PSYCHOSOCIAL COUNSELORS**<br>**Philipp** (*white*, cis man, psychosocial counselor) about mental healthcare providers refusing to treat refugees or racialized service users: “Yes, there

				might also be some racist stereotypes among the practitioners, but the main issue is probably that they don't find a treatment offer and people say things like: 'I don't really know much about refugees' or 'That's too complicated. I don't have any capacities for this at the moment,' or they don't say anything at all."
	**b) Institutional Discrimination**	Interpretation services and time constraints	Interviewees describe challenging or failed situations of communication with service users based on lack of interpretation services or time constraints.	**MENTAL HEALTHCARE PROVIDERS**<br><br>**Rebecca** (Black, cis-woman, mental healthcare provider), reporting on an incidence in inpatient psychiatric services: "They didn't call in the interpreting service. They somehow called in the nursing staff, who spoke Italian. We had a Spanish-speaking nurse who then had to translate. It's absurd when you think about it. […] But they still gave him some medication, and I thought, 'On what basis?' when there was no real conversation […]. [And if a nurse interprets], it's still a role shift for the nurse. I don't think throwing someone in there is a good idea. […] It's not really an option, even if this nurse could translate perfectly. That's not why she's there. There's a shift in contact, and you can see that this psychiatric nurse is there to provide support. When the colleague was there, the patient had a contact person. Otherwise, he was lost. This nurse could now provide that support, which was really helpful for the patient. But it's not a sustainable solution […] I don't think that works on many levels. I think it's important to have an initial consultation in the language the patient can speak, which can be tricky with smaller languages that often switch to a colonial language, for example."<br><br>**Jan (***white*, cis man, mental healthcare provider) on how language barriers in clinical encounters**:** "Exactly, that was another point. I had already thought about it beforehand because […] because it is definitely relevant and certainly contains discriminatory elements. There's no possible one-size-fits-all solution, so ultimately, the patients are required to bring an interpreter with them. In principle, if they can't speak German at all, or at least English, depending on the doctor, they won't get an appointment."<br><br>**Peter** (*white*, cis man, mental healthcare provider) on therapy offers for non-

				German-speakers on his ward: “We don’t […], we don’t offer services for people who do not speak German well, because […], it is caused by the way that [form of psychotherapy] is conceptualized. Those are therapy sessions, that demand a lot of preparation and follow-up work, including working with materials in the German language. Sometimes we have patients that need support due to a slight cognitive disability for example when filling out work sheets. Of course, we attend to that and if it is necessary we will read the sheet together or read to them so they can manage it somehow. For foreign languages, we don’t really have anything to offer. No. Unfortunately not.”<br><br>**Jan** (*white*, cis man, mental healthcare provider): “Exactly, there was one situation that stuck with me a bit: I was in the room where the medical assistants were sitting, one of them was on the phone and it’s a very unpopular job with them, because they get a lot of calls, and she was very annoyed […] when I came over, spoke to me and said something like: 'Yes, today it’s so bad. There are always people calling who don’t really speak German. What do they want here anyway? And they all want […] – I got the impression – they just want to get a certificate of incapacity for work.’ And there were two elements to that: on the one hand, she was very negative about the fact that there are a lot of people calling who don’t speak German or have problems speaking fluent German, and the other component, which was certainly more of an insinuation, was that they all just want a certificate of incapacity for work. […] There’s probably a lot of frustration involved. Communication problems can also hold up the daily work routine, can make phone calls take a long time.”<br><br>**PSYCHOSOCIAL COUNSELORS**<br><br>**Philipp** (*white*, cis man, psychosocial counselor): “We often get feedback from clients that the doctors on duty in the psychiatric ward say there’s no language mediation for inpatient treatment and that clients should stay with us for outpatient treatment because we do have interpretation services. Or that they should look for native-speaking psychiatrists, which, if they now

				need inpatient treatment due to self-endangerment or acute, acute, high symptomatology, acute need, is discrimination because they don't have the same access to healthcare."
		Room assignment, admissions and IT systems	Interviewees describe instances of discrimination that follow from room assignment practices or admission and IT systems.	**SERVICE USERS**<br>**Leonie** (*white*, trans woman, service user) on her experiences during an psychiatric inpatient stay: "Since I am a trans person, what was a big problem for me there and what also – yes – had a significant negative impact on the recovery process or the progress, was or is simply that Hospital C is purely all […], all […] that it is run in a purely binary way, and accordingly I was assigned to a room according to my gender, which is what it says on my identity card, so I was assigned to a room with a man and that was – yes – not so good for me, so I had to be in a room with a man most of the time, most of the day."<br>**HEALTHCARE PROVIDERS**<br>**Matthias** (*white* and Russian-German, cis man, mental healthcare provider) on his experiences of admitting trans patients on his ward: "As a registrar, I have a waiting list for my ward. If there's a trans person on the waiting list, there's always the question of what kind of room the trans person will be put in. This is because in psychiatry […], we still work in a way that's based on binary gender patterns. There are women's rooms and men's rooms. The senior physicians [here] are both a bit more 'critical' of the whole trans issue. This makes it sometimes a bit difficult to admit trans patients to my ward."<br>**Matthias** (*white* and Russian-German, cis man, mental healthcare provider): "I have a patient [in the IT system] and the deadname is visible and not the name I actually want, and I have no way, no column where I can enter pronouns, and even if I did, I'm pretty sure it would be complicated for the nursing staff, but also for medical colleagues and other multi-professional staff, to address people correctly."

		Lack of competencies among mental health providers, adapted treatment options, and environments	Interviewees describe how mental healthcare providers lack the competencies (knowledge, skills) to address challenges related to structural discrimination, or how treatment options and physical treatment environments are not adapted to the needs of marginalized service users.	**SERVICE USERS**<br><br>**Alex** (*white*, trans* man, service user): "I once flipped through a few magazines. There were very, I'll say, homo […], homophobic and transphobic things in there. They were Christian – I'll say – psycho magazines […] There were several posters on the walls […] from a Catholic news portal […] There were some really nasty things on them […] I felt very uncomfortable."<br><br>**MENTAL HEALTHCARE PROVIDERS**<br><br>**Rebecca** (Black, cis-woman, mental healthcare provider) on providing therapy to Black service users: "I have to say, there are a lot of manuals for behavioral therapy and I always say […] I always put a trigger warning on them because they are written for specific people. So I have one now with, I don't know, Mr. Mouse and Mr. Klaus. I don't know, the dialogs are always between the two of them and I always say: 'Okay, this isn't necessarily tailored to Black people, but read it in the sense of […].'"<br><br>**Jan** (*white*, cis man, mental healthcare provider) on why he does not ask about experiences of racism in his consultations: "In order to perhaps be able to alleviate the patient's burden to a certain extent, although even then it would of course be somewhat important to know: If I address this, how do I deal with it, what can I offer the patient? […] So there's no point in exploring it if I don't have a concept that I can work on with the patient."<br><br>**Matthias (***white* and Russian-German, cis man, mental healthcare provider) about their own competencies**:** "Currently I don't feel, in regard to that topic, not […], only trained to a certain degree to deal with it, especially in […] especially in regard to racism, where I feel that I am certainly making mistakes and that I have assumptions that I am not noticing myself […] and the question is, if I open the topic but then don't have anything to offer, I think that is a shame."<br><br>**PSYCHOSOCIAL COUNSELORS**<br><br>**Lou (***white*, no gender category, psychosocial counselor) on treatment refusals by mental healthcare providers based on lack of competencies:

				“[They say]: ‘We can’t do this. We can’t do that,’ and so people who are really specialists but still say: ‘We don’t have the competencies to look after this person and we’d rather accept that no one will look after them now.’”
**2. Strategies and Reactions**	**a) Service users’ strategies and reactions:**	Confrontation and asking for support	Interviewees describe confronting perpetrators after incidents of discrimination or asking others (staff, other patients) for support.	**SERVICE USERS**<br><br>**Layla** (Arab, nonbinary, service user), reporting about an incidence on an inpatient psychiatric ward: “I started by saying that I’m nonbinary, and then she said, ‘No, that’s not for us.’ She’s young, by the way, and then she said: ‘That’s […] it’s going to be too difficult, just so you can prepare yourself, it’s going to be too difficult. You’ll be misgendered and that […] because that doesn’t happen here,’ and then I just smiled like this and then I said: ‘Yes, by the way, the last time I was hospitalized, they didn’t call me ‘woman.’”<br><br>**Johannes** (*white*, trans* man, service user) on an experience during an inpatient stay: “It happened […], during my first stay here in [major German city], that I once walked past the nursing staff’s room and, and I had spoken to them briefly, I don’t know, that I was then addressed in the wrong way, with the wrong [name and gender], and that was initially like ‘What just happened?,’ because I hadn’t been addressed like that for three years, and […] it had never happened to me before or never been a question, and that was also the person from the nursing staff who had access to my files, and I know that the old name is still in my files from five years ago […] and then I asked them for a chat and said […]. So I tried to be nice and polite, I was polite too, but I told her it must not happen again, because if it came out, then I would switch wards, then I would just leave, no matter how shitty it was, I wouldn’t put up with it anymore and that was also the case, then I would feel unsafe, because there are also people on the ward and other patients. I don’t want to […], I just don’t want to explain it anymore. Like that. And that’s also what I would do today, if it were now […], I wouldn’t accept it nowadays, I would just leave, no matter what. But back then I was desperate and had no idea.”

		Withdrawal and selective narration	Interviewees describe communicative and behavioral strategies to prevent further instances of discrimination and to protect themselves from negative reactions and further harm.	**SERVICE USERS**<br><br>**Toni** (*white*, trans* and nonbinary, service user): “So after the suicide attempt, I actually mentioned being trans *at the end* and also the conversation in [German city], I also mentioned it at the end, just because otherwise there can be a bias and people don’t really listen, and if you mention it at the end, there’s a better chance that they’ve listened at the beginning, that – I don’t know – it doesn’t get pushed aside somehow.”<br><br>**Momo** (POC, cis woman, service user): “I had to deny one part of my identity or not talk about it and that is how I went into the day clinic in the second week, because I thought: ‘Okay, I’ll take fifty percent with me and I’ll deny the other half, because I don’t have any other options in Germany.’”<br><br>**Johannes** (white, trans* man, service user): “So I knew which areas I could touch on and which I couldn’t, and it was just that I didn’t touch on anything to do with trans, because I unfortunately had also experienced that in the outpatient therapy that I was doing at the same time, that the therapist […] asked me questions that gave me the impression that she didn’t support it at all. So […] and then it wasn’t brought up in the clinic, […] so I didn’t bring it up because I knew it wouldn’t work.”<br><br>**Alex (***white*, trans man, service user) on their treatment experiences: “I said a lot of nonsense at the beginning, just to fit in with this cliché, and I’m really glad that [the doctor] managed to build up trust. Yes, that was or is a very positive experience, it […] Yes, so it’s really a very basic thing that I don’t have to expect any negative consequences for any things that I disclose, i.e. for telling the truth, i.e. how I feel, and for behaving the way I normally behave, i.e. something very basic.”<br><br>**Johannes (***white*, trans* man, service user): “I’ve often found that therapy can be very helpful and that I’ve usually met with openness and acceptance and help with most issues when I’ve addressed them. So. But I would never bring up the trans thing in therapy nowadays, because I’ve had shitty experiences with it, and I’m waiting for it now, so I […], I first have to hear

				something from the person treating me that is either queer-friendly or green, sustainable, something that goes in that direction, and if I don't hear something like that, then I don't bring it up, even if I like the person. But I do need that […] I need some kind of indication that they might not react like shit in order to address the trans thing somehow."<br><br>**Mika** (Arab, cis man, service user) explains his attitude towards past occurrences of racial slurs: "So I've had the experience since I've been in Germany, even with these treatments and hospitals. I know from the outset what I'm in for, that I'm a shitty […] mafioso or something similar […], but [I] know it now, I'm aware of it."
	**b) Mental healthcare providers' strategies and reactions:**	Trivialization	Instances of discrimination are trivialized or their effects downplayed.	**HEALTHCARE PROVIDERS**<br><br>**Jana** (white, cis-female, mental healthcare provider), explaining why she thinks that asking trans people about their gender is not discriminatory: "So a person like that [a trans person] will certainly have to explain more often what their gender is, how they should be addressed, what particularities might need to be taken into account. But now, for example, a patient who has allergies also has to explain what allergies they have and what needs to be taken into account."<br><br>**Peter (***white*, cis man, mental healthcare provider) on the question whether there was discrimination in mental healthcare based on language or country of origin**:** "I believe […] that this exists, but […] it is clinically […] not so often relevant."<br><br>**Ruben (**Latinx and POC, cis man, mental healthcare provider) describes how his colleagues reacted when he told having experienced racism by a colleague: "Yes, well, I was […], they were a bit like horrified, but somehow it was like: 'Yes, there are stupid people everywhere,' and 'She's the one with the problem. Don't worry about it.'"<br><br>**Jan** (white, cis man, mental healthcare provider) commenting on the challenges of receiving an appointment for non-native German speakers:

				"Even if you have perfect language skills, I think it can happen that, depending on the mood of the person, you either get an appointment or are turned away. Sometimes I think it just depends on their mood, I don't know."
		Defensiveness	Interviewees related situations in which others reacted to descriptions of discrimination with defensiveness or interviewees becoming defensive during the interview.	**SERVICE USERS**<br><br>**Layla** (Arab, nonbinary, service user) describing staff's reaction to their complaint about discrimination: "[They said:] 'Everything you said sounds like your problem. You're sensitive,' and because of my disorder and me […] that it's just my problem and not theirs. Exactly. And that was the worst experience I've ever had."<br><br>**Alex (***white*, trans man, service user) describes how he tries to protest against transphobic magazines and posters at the psychiatric ward: "When I spoke to a therapist about it after a while, after two weeks or so, he just told me what nonsense I was talking about and that it was some posters, I shouldn't worry about it and so on. I wasn't responsible for the furnishings here and he just slammed the door of his office in my face, really."<br><br>**MENTAL HEALTHCARE PROVIDERS**<br><br>**Julia** (*white*, cis woman, mental healthcare provider) criticizes a patient's sense of entitlement. Asked whether she would say the same about a German patient, she explains: "But of course it depends on the […], on the person […] So. Yes, the […], this personal relationship with the individual patient. I think that is decisive. Yes, I also have German patients who get by like that, so where I […], […] Like that. But there […], then I would never have had such negative feelings [as in the case of the non-German patient]."<br>**Interviewer**: "And where does that come from? […]"<br>**Julia**: "Maybe, because sometimes you don't understand the cultural background anyway. Sometimes you don't understand it at all. Like this."
		Acknowledgment	Interviewees acknowledge the impact and pervasiveness of	**MENTAL HEALTHCARE PROVIDERS**

		and support	structural discrimination and describe strategies to support marginalized service users or colleagues.	**Jan** (white, cis-male, mental healthcare provider), asked about the situation for trans service users on his ward: “So I do think that you could […] that one could do more. Yes. I still have to […], I also remember, when I think about it, even with nurses and so on, it sometimes causes great uncertainty to even ask which patients can somehow be put in a room, even in double rooms. Of course, the question comes up. That creates uncertainty. There have also been occasional jokes between nursing staff and doctors about people who have changed their gender identity several times. That also happened. I can’t literally remember now, but it’s just that it was made a bit of a joke because they probably didn’t know how to deal with it.”<br><br>**Peter (***white*, cis man, mental healthcare provider), on his ward’s attitude towards trans service users: “Another thing is the practical – let me put it this way – […], the, the […], the interactional […], dealing with people. How are they addressed? And that’s why we sat down as a team – it wasn’t that long ago – six months, nine months ago and decided, so to speak, that we would simply act in accordance with the guidelines and ask how someone would like to be addressed, and we’re actually managing relatively well with that now.”<br><br>**Ruben (**Latinx and POC, cis man, mental healthcare provider), on his ward’s attitude towards discrimination: “People are misgendered or something like that, I […], sometimes it’s by mistake, because it’s also not in the file somehow, that it’s actually nonbinary or not with woman or only with woman or with man or […] and then, if, if misgendering happens, then here at least the outpatient clinic, from experience, they apologize, they apologize: ‘Okay, and then I'll make a note there and then we’ll stick to it,’ exactly.”<br><br>**Jan** (*white*, cis man, mental healthcare provider), talking about his lack of knowledge on racism: “It scares me a bit, because I think I also happened to read an article a week or so ago in the national German weekly newspaper A, now, where it’s also about discrimination in psychotherapy […] and that it’s not discussed enough and is very central for some people. That’s why you get scared, because you don’t know whether you’ve missed something

				really relevant for the treatment of the patient." <br>**Ruben (**Latinx and POC, cis man, mental healthcare provider), commenting on the prevalence of racism**:** "And actually, I think it's also good for employees who do this kind of training on racism, but also with examples of what is […], what happens in everyday life, and I mean […], we're all affected by it. We can be non-racist, but somehow culturally or linguistically there are always these jokes, words or something that are actually racist, and we don't perceive them that way because we just don't know it." <br>**Jan (***white*, cis man, mental healthcare provider), on his ward's response to racism: "There have been cases where patients have been racially insulted by patients and then we had to intervene, and if we had the opportunity, the patients who made racist insults were also discharged."
**3. Effects of discriminatory practices**	**On service users**	Emotional Response	Interviewees describe different emotional responses of service users to experiencing discrimination.	**SERVICE USERS** <br>**Toni** (*white*, trans* and nonbinary, service user): "But at the latest after her statement I was just so angry and I just didn't want to interact with this person anymore and I just didn't want to answer the person or anything because I was so […] I just thought to myself: Nope." <br>**Leonie (***white*, trans woman, service user), talking about her response to an instance of discrimination: "So – yes – I wasn't feeling well anyway and I didn't feel strong enough to have any more discussions like that." <br>**Alex (***white*, trans man, service user), narrating an interaction with a therapist: "So it was extreme. It wasn't just unsatisfactory, I was really miserable because I was […], it [my wish of being pregnant as a trans man] annoyed her all the time. Yes, she was disgusted by me. I really kind of felt that way. She didn't say it, but I'm really sure that she was somehow disgusted." <br>**Mika (**Arab, cis man, service user) answering to the question how he reacted to discriminatory remarks: **Interpreter**: "Unfortunately, I can't do anything about it. So it's […],

				makes me sad, but I can't defend myself against it. I don't."<br>**Mika**: "I can't change that. And I've even noticed that if I complain or say something, the doctor there or the nurse does shit to me afterwards. It also happened, but not in the form of a punishment, but with strict treatment."<br><br>**Momo** (POC, cis woman, service user): "I came out of the doctors' visit and on the first day I was so shocked because I thought: Shit, now I'm here because I'm not feeling well mentally and I'm getting into a situation like this, now racism, which is also something that concerns me, and I've got someone who's in a position of power. What do I do now? Do I mess with him? Where do I complain? And if I argue with him or complain, what do I have to fear."<br><br>**PSYCHOSOCIAL COUNSELOR**<br>**Philipp** (*white*, cis man, psychosocial counselor) commenting on the effects of chronic discrimination: "The main reaction is the feeling: 'This is happening to me […],' and/or this question: 'Why is all this bad stuff happening to me?,' i.e. with a history of traumatic violence and so on, and then small experiences of rejection, experiences of discrimination, which, so to speak, hit this notch and cement this feeling of the bad world. 'It always happens to me.'"<br>**Lou (***white*, no gender category, psychosocial counselor): "Very often there's also a problem when people are in hospital somewhere and can only actually be in single rooms because the hospital doesn't really have the heart to accommodate them somewhere else, and now […], you might think it would be nice to have a single room, but people are often actually very sad about it and say: 'Why am I being excluded again? Why can't I be in the room with other women?'"
		Changes in mental healthcare	Interviewees describe how service users change their mental healthcare service	**SERVICE USERS**<br>**Layla** (Arab, nonbinary, service user), asked if they would go to a mental

		utilization	utilization in reaction to having experienced forms of discrimination.	healthcare service again: "So, if I had two options, that I would go to psychotherapy and experience discrimination again […] but there's also a chance that I would be better, OR that I would stay the way I am, or even worse, but without experiencing discrimination, and I just chose the second one. Yes."<br><br>**Toni (***white*, trans* and nonbinary, service user) commenting on the effects of having experienced discrimination in mental healthcare: "For one thing, it definitely makes you even more afraid to go to a doctor, i.e. to therapy, or at least to go to hospital, because you immediately have this thought: 'Some bad experiences have already happened. What if it happens again?' […] The other thing is that in both cases I was in absolute crisis situations and at the absolute lowest point and would have needed help, but I didn't get it, and I think things would certainly have gone much better if I had gotten help at that moment."<br><br>**Alex (***white*, trans man, service user) on his reluctance to access mental healthcare for fear of discrimination: "So over the years it's gotten worse and worse […]. And I didn't want to get any more help at the clinic the whole time, because I thought: 'It won't work out anyway. It's always the same old issue, it's like that everywhere,' because I've also heard from other people how bad it was in clinics, I mean, I also work with trans people. I've heard enough."<br><br>**MENTAL HEALTHCARE PROVIDERS**<br><br>**Johannes (***white*, trans* man, service user) on the effects of transphobic microaggressions in mental healthcare: "But also as a microaggression from the system somewhere, from a person, actually from a person from whom you seek help, a supposed expert on the topic, which is also recorded somewhere as a bad experience along the lines of: 'They simply can't help me in psychiatry when it comes to this topic. On the contrary: I'm actually being judged,' which I think is incredibly problematic in this respect."<br><br>**Rebecca (**Black, cis-woman, mental healthcare provider), on users' strategies to navigate discrimination: "So. now in outpatient psychotherapy, when I look at my colleagues' clients, they just look different from mine, so

				I really […], I have a lot of requests from POCs and also from queer people. I have a – yes – , […] a lot of requests from white queer people. Exactly, and I have a lot of people of color who come knocking.”
		Deterioration of mental health status	Interviewees describe the deterioration of service users' mental health in the context of having experienced discrimination within mental healthcare.	**SERVICE USERS**<br>**Alex** (*white*, trans man, service user) describes the effects of being discriminated against and misgendered in mental healthcare: “So, the first two weeks were total hell, and I just wanted to get away, and I developed some really paranoid thoughts. […] I didn't know these thoughts of myself. I didn't recognize myself. Sometimes I was really scared, but otherwise I thought […] I still knew somehow that it couldn't be real. But then somehow it took on strange proportions for me because of this treatment, and I became very suspicious.”<br>**MENTAL HEALTHCARE PROVIDERS**<br>**Matthias** (*white* and Russian-German, cis man, mental healthcare provider) on the health effects of discriminatory treatment: “But that also means that trans patients are ultimately on the waiting list for longer, they get sicker and then actually come to us on the ward in a more acute condition and they don't get the same kind of healthcare as people who don't identify as trans.”

Table 4 *Definitions and exemplary quotes for subthemes*
